# Modulation of Item and Source Memory by Auditory Beat Stimulation: A Pilot Study With Intracranial EEG

**DOI:** 10.3389/fnhum.2018.00500

**Published:** 2018-12-11

**Authors:** Marlene Derner, Leila Chaieb, Rainer Surges, Bernhard P. Staresina, Juergen Fell

**Affiliations:** ^1^Department of Epileptology, University of Bonn, Bonn, Germany; ^2^Department of Neurology, University Hospital RWTH Aachen, Aachen, Germany; ^3^School of Psychology, University of Birmingham, Birmingham, United Kingdom

**Keywords:** binaural beat, monaural beat, associative memory, hippocampus, rhinal cortex, EEG phase locking

## Abstract

Auditory beat stimulation is an upcoming technique for non-invasive brain stimulation. Its influence on mediotemporal regions and memory processes has not yet been thoroughly investigated. A recent study suggests that auditory beats are able to alter intracranial EEG (iEEG) power and phase synchronization. 5 Hz binaural beat stimulation increased temporo-lateral phase synchronization, while 5 Hz monaural beat stimulation decreased mediotemporal synchronization. Based on the relevance of phase synchronization for memory operations, we hypothesized that 5 Hz binaural beat stimulation enhances, while 5 Hz monaural beat stimulation decreases long-term memory performance. We analyzed data from presurgical epilepsy patients with implanted depth electrodes in the hippocampus and rhinal cortex. 5 Hz monaural and binaural beat vs. control stimulation was applied while patients performed an associative learning task involving item and source recognition. We evaluated behavioral effects for item (hits minus false alarms) and source memory (correct minus incorrect) and the impact of auditory beats on iEEG power, rhinal-hippocampal phase synchronization and inter-trial phase locking. A three-way repeated measures ANOVA (encoding/retrieval, item/source, monaural/binaural/control) revealed a main effect of stimulation (*p* = 0.03) and a linear effect in the expected direction: binaural > control > monaural (*p* = 0.036). Both monaural and binaural stimulation were associated with increased phase locking of 5 Hz oscillations within rhinal cortex. These phase locking increases, however, corresponded to reverse phase shifts. Our data suggest that binaural vs. monaural 5 Hz stimulation increases vs. decreases long-term memory performance. These behavioral effects appear to be related to reverse phase shifts within rhinal cortex.

## Introduction

The number of patients suffering from memory disorders, like Alzheimer’s disease, is continuously rising (Wimo et al., 2003). This makes the need to explore new therapeutic approaches for memory enhancement imperative. One promising approach is deep brain stimulation of mediotemporal regions, such as rhinal cortex and hippocampus, which are crucial for long-term memory. In spite of some promising reports (for reviews see, e.g., Lee et al., 2013; Sankar et al., 2014), findings are thus far inconclusive. Even under application of similar protocols, opposite outcomes regarding memory performance have been reported (e.g., Suthana et al., 2012; Jacobs et al., 2016; Hansen et al., 2018). Apart from this controversy, deep brain stimulation using intracranial electrodes is a highly invasive procedure and long-term application is prone to complications.

Recently, several non-invasive brain stimulation methods have been reported to be able to influence mediotemporal regions (for an overview, see Polanía et al., 2018), including transcranial electric stimulation with temporally interfering fields (Grossman et al., 2017) or pulsed transcranial ultrasound stimulation (Tufail et al., 2010). Another upcoming technique, whose impact on memory processes has not yet been thoroughly investigated, is stimulation with auditory beat signals (e.g., Chaieb et al., 2015; Hommel et al., 2016). Auditory beats are amplitude modulated tones with modulation frequencies in the range of typical EEG rhythms. For instance, beat signals can be constructed by superposing two sine waves with nearby frequencies. Beat stimulation is either applied by presenting amplitude modulated beat signals to one ear or both ears (monaural beats), or by presenting the original sine waves separately to each ear (binaural beats). In this more frequently investigated case beat perception results from the responses of phase-sensitive brain stem neurons (Wernick and Starr, 1968).

Until now, results of the few studies addressing the influence of beat stimulation on cognition are divergent (see e.g., Chaieb et al., 2015; Chaieb et al., 2017). Regarding the electrophysiological effects of binaural beat stimulation, alterations of EEG power (e.g., Gao et al., 2014; Ioannou et al., 2015) as well as beat-induced phase delays (Schwarz and Taylor, 2005; Ross et al., 2014) and changes in interregional phase synchronization (Ioannou et al., 2015) have been reported. Based on intracranial recordings in presurgical epilepsy patients, we recently demonstrated that monaural and binaural beat stimulation caused specific changes in both intracranial EEG (iEEG) power and phase synchronization even in mediotemporal regions (rhinal cortex and hippocampus) (Becher et al., 2015). In particular, we observed that 5 Hz binaural beat stimulation was related to an increase of temporo-lateral phase synchronization, while 5 Hz monaural beat stimulation was associated with a decrease in mediotemporal phase synchronization. Since phase synchronization has been suggested to support long-term memory operations via facilitating neural communication and synaptic plasticity (Fell and Axmacher, 2011), we hypothesized that binaural beat stimulation at a frequency of 5 Hz may enhance, while monaural beat stimulation at the same frequency, may decrease long-term memory performance.

To investigate this hypothesis, in this pilot study we applied monaural and binaural beat stimulation at 5 Hz to presurgical epilepsy patients during an associative learning task (Staresina et al., 2012). This task has been shown to particularly depend on rhinal and hippocampal operations (Staresina et al., 2013) and allowed us to distinguish between memory for items and memory for associated source details. Effects of beat stimulation were compared to effects of a control stimulation condition comprised of a pure sine wave having a frequency identical to the beat carrier frequency. For each patient, we alternately stimulated during encoding and retrieval periods to be able to examine the impact of the auditory beat signals on the different stages of memory processing. In addition to evaluating behavioral effects, we analyzed the impact of monaural and binaural beat stimulation on electrophysiological patterns within rhinal cortex and hippocampus in terms of iEEG power, rhinal-hippocampal phase synchronization and inter-trial phase locking.

**FIGURE 1:**
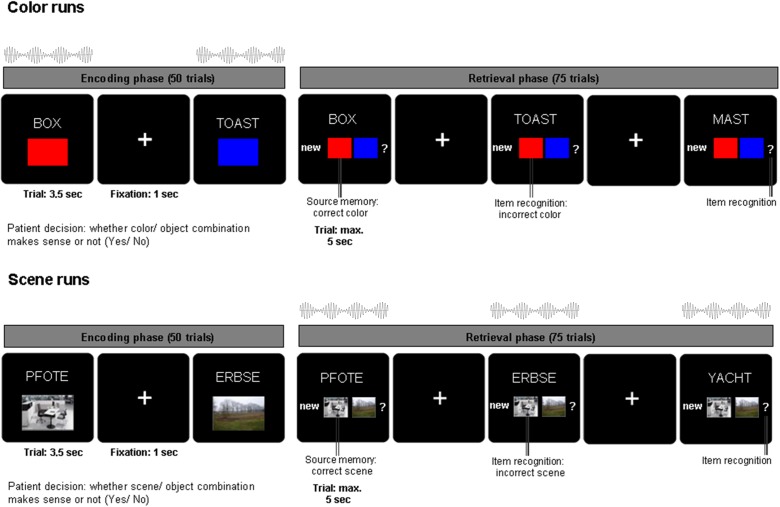
Associative learning paradigm and beat stimulation. During encoding, nouns were presented in combination with one of two colors or one of two scenes for 3.5 s and patients indicated whether the combination was plausible or not. During retrieval, the previously presented nouns along with previously unstudied nouns were shown for a maximum of 5 s. Patients indicated their memory of the noun color/scene association with one of four possible responses: (i) new noun, (ii) old noun associated source one, (iii) old noun associated source two, (iv) old noun but unable to remember association. Auditory beat stimulation was presented either during the encoding (color runs, top) or during retrieval phase (scene runs, bottom).

## Materials and Methods

### Patients

Fifteen presurgical epilepsy patients with depth electrodes implanted in the medial temporal lobe participated in this pilot study (8 females, mean age 36.3 ± 11.4 years). The study and all experimental procedures were approved by the Ethics Committee of the Medical Faculty of the University of Bonn. All patients gave written informed consent in accordance with the Declaration of Helsinki. Presurgical evaluation revealed hippocampal seizure foci in eight patients, temporo-lateral foci in four patients, and frontal foci in three patients. In six patients the seizure focus was in the left hemisphere, in seven patients it was in the right hemisphere, and in two patients the foci were bilateral.

### Experimental Paradigm

Patients were asked to perform an associative memory task (see Figure 1). During the encoding phase of the task, stimuli consisting of 50 German nouns (per run) were presented on a computer screen in combination with a color patch (red/blue) or a scene image (office/nature) for 3.5 s. After each trial, there was a jittered inter-trial interval of 700–1300 ms (mean = 1000 ms) during which a fixation cross was presented. Nouns were displayed in white uppercase letters and centered on a black background. The associated source (a color or scene, depending on the experimental block) was presented in a 200 × 300 pixels frame positioned 160 pixels underneath the noun. For each trial, patients were asked to indicate with a button press whether the association between the color/scene and noun was plausible or not. The retrieval phase of the task started after a 1 min break. During the retrieval phase, the 50 nouns previously presented during the encoding phase were shown again together with 25 previously unstudied nouns. Underneath the noun, the response options “new” (i), the two possible color/scene sources (ii, iii) and a question mark (iv) were shown. Each trial was displayed for a maximum of 5 s. Patients were instructed to indicate whether they remembered the previously shown noun and the corresponding color/scene associations. Patients were asked to respond with one of four possible decisions: (i) new (the noun has not been previously shown during the encoding phase), (ii) old [the noun has been presented in combination with source 1 (e.g., red/office)], (iii) old [the noun has been previously presented with source 2 (e.g., blue/nature)], (iv) old [previously presented noun but unable to remember the association (?)]. Thus, patients indicated with one button press not only whether they remembered the word, but also whether they remembered the combination of the word and the associated color or scene. Patients were encouraged to respond to each trial as accurately and as quickly as possible. In each experimental run (consisting of 50 encoding trials and 75 retrieval trials), only one source category (color or scene) was used. Nouns were randomly chosen without overlap between experimental runs out of a list of 450 nouns. Color and scene associations were also randomly chosen for each run.

### Auditory Beat Stimulation

Across six experimental runs, auditory beat stimuli or the control tone were presented to the patients either during the encoding phase (for color source runs only) or the retrieval phase (for scene source runs only; see Figure 1). This design was chosen in order to reduce a possible spill-over of modulation effects from the runs with stimulation during encoding to the runs with stimulation during retrieval. In other words, if beat stimulation would influence associative word-color networks during encoding, the risk of a spill-over to a run with stimulation during retrieval was intended to be minimized by employing scene associations (recruiting word-scene networks), and vice versa. The stimulation conditions were as follows: binaural beats (5 Hz), monaural beats (5 Hz), control tone (220 Hz – pure sine wave, no beat). The order of stimulation conditions was randomized and counterbalanced across patients. Auditory beat stimulation was presented at stimulus onset for the duration of each trial (encoding: 3.5 s, retrieval 5 s).

Auditory beats were generated using two sine waves of nearby frequencies. In order to produce 5 Hz beats the frequencies of 217.5 and 222.5 Hz were employed. Monaural beats are amplitude modulated acoustic signals resulting from the physical superposition of two sine waves. In our study this superposed beat signal (217.5 Hz sine wave plus 222.5 Hz sine wave) was presented to both ears simultaneously. In the case of binaural beats, one sine wave (e.g., 217.5 Hz) was presented to one ear, while the other sine wave (e.g., 222.5 Hz) was presented to the opposite ear. The alternating phase shifts due to the frequency mismatch between the two sine waves generate the binaural beat percept. The interaural frequency difference corresponds to the beat frequency that is perceived (in our case 5 Hz). In addition, a control tone of 220 Hz (pure sine waves without amplitude modulation) was presented to both ears simultaneously. All auditory stimuli were applied with an average sound pressure level of 75 dB and delivered through over-ear headphones. Stimuli were created using the NCH Tone Generator (NCH software, Canberra, ACT, Australia) and presented using Presentation^®^ Software (Version 16.5, NeuroBehavioral Systems Inc.).

### Behavioral Data Analysis

For behavioral data analyses, we defined two memory effects of interest: (i) an adjusted item memory effect given by “the probability of a hit minus the probability of a false alarm”; i.e., (correct old responses to studied items / all responses to studied items) – (incorrect old responses to new items / all responses to new items) and (ii) an adjusted source memory effect given by “the probability of a hit minus the probability of a failure” (excluding “unsure” responses); i.e., (correct source decisions – incorrect source decisions) / correct old responses to studied items. Statistical analyses were performed using repeated-measures ANOVA (with Huynh-Feldt correction) and subsequent paired two-tailed *t*-tests. Two patients were excluded from the behavioral analysis due to missing data (i.e., 13 patients were included). One patient performed only three color runs, and one patient chose in the case of hits only the question mark response during one of the runs. In this instance source memory could not be reliably accessed.

### IEEG Recordings and Artifact Rejection

Intracranial EEG was recorded from depth electrodes implanted in the medial temporal lobe (eight cylindrical platinum contacts, diameter: 1.3 mm; length: 1.6 mm). In all patients the electrode placement was ascertained using magnetic resonance images which were mapped to standardized anatomical atlases (Duvernoy, 1988). IEEG was referenced to linked mastoids and recorded using a sampling rate of 2048 Hz. Only iEEG recordings from patients with unilateral seizure onset zones were considered for further analysis (i.e., the two patients with bilateral foci, who were included in the behavioral analysis, were excluded from iEEG analyses) and the contacts from the non-pathological hemisphere were included in the analysis. BrainVision Analyzer (Version 2.0, Brain Products) was used for electrode selection and rejection of epileptiform, movement or technical artifacts. Artifact rejection was performed via visual inspection and 22.4% of all trials were discarded.

For each patient one rhinal and one hippocampal electrode contact was chosen. The selection of contacts was based on the following three criteria: (i) contacts from the hemisphere contralateral to seizure onset zones (ii) structural MR information (iii) average event-related potentials elicited during the recognition phase. Trials were segmented from -1000 to 3000 ms with regard to stimulus onset and baseline corrected by subtracting the average of the baseline interval defined from -200 to 0 ms. Event-related potentials were averaged across correctly classified old and new items. Based on previous studies, the rhinal (RH) contact was defined as anatomically located within rhinal cortex and exhibiting the largest mean amplitude of the negative component between 250 and 750 ms. The hippocampal (HI) contact was defined as located within anterior or middle hippocampus and showing the largest mean amplitude of the positive component between 350 and 850 ms. These event-related potentials correspond to the so-called anterior medial temporal lobe N400 component and the hippocampal P600 component. These memory-related components have been consistently reported in many studies (AMTL N400: e.g., Guillem et al., 1995; Nobre and McCarthy, 1995; Grunwald et al., 1999; Fell et al., 2008; Staresina et al., 2012, 2013; hippocampal P600: e.g., Guillem et al., 1995; Ludowig et al., 2008; Fell et al., 2008; Staresina et al., 2012, 2013). Datasets without electrode contacts located in the target zones (RH or HI) or without pronounced event-related potentials were discarded. After considering these criteria datasets from seven patients could be used for further iEEG analysis. For one of these patients no rhinal channel was available and one patient performed only color source runs. This resulted in the following number of contacts for the different experimental conditions: (i) color source, auditory stimulation during encoding phase: six rhinal contacts and seven hippocampal contacts; (ii) scene source, auditory stimulation during recognition phase: five rhinal contacts and six hippocampal contacts.

### Quantification of Phase Synchronization, Phase Locking and Power Values

Intracranial EEG phase characteristics and power values were calculated for the encoding phases of the color runs and for the retrieval phases of the scene runs, i.e., for trials where auditory stimulation was applied. All trials in the encoding phases, and old/previously studied trials in retrieval phases were segmented from -1000 to 2800 ms with regard to stimulus onset and sorted according to the different stimulation conditions. The average number of trials per patient in the encoding phase was 39.5 (23–48) and the average number of old/previously studied trials in the retrieval phase was 38.9 (28–47).

Intracranial EEG signals were filtered at 5 Hz by continuous wavelet transforms with Morlet wavelets of five-cycle length. In order to avoid edge effects the resulting signals ω were cut to the interval from -200 to 2000 ms. Power values [Pow_j_ = abs(ω_j_)^2^ = Re(ω_j_)^2^ + Im(ω_j_)^2^], phase values {φ_j_ = arctan[Im(ω_j_) / Re(ω_j_)]} and phase differences between rhinal and hippocampal contacts [Δ_j_ = φ_j_(RH) - φ_j_(HI)] were extracted for each time point j of each trial. In the following, we will refer to rhinal/hippocampal phase as the time dependent phase value of the band-pass filtered iEEG recorded from within rhinal cortex/hippocampus.

Inter-trial phase locking and phase synchronization were calculated based on the circular variance of phases. Phase locking/synchronization is given by the length of the mean complex phase/phase difference vector across all trials for each condition (Lachaux et al., 1999). As it can be expected that the circular variance depends on the number of values considered for its calculation, the same number of trials was randomly chosen from each condition for each patient by subselecting a random portion of the conditions with more trials to match the condition with fewest trials (range: 23–43 trials). Power, phase locking and phase synchronization values were baseline normalized by dividing them by the average value from the -200 to -100 ms interval (i.e., the baseline) across trials separately for each condition and subject (i.e., the baseline level corresponds to the value 1).

It should be noted that the calculated phase locking values are based on inter-trial phase concentrations with regard to the onset of both the beat stimuli and the nouns plus associative stimuli (since both were delivered at the same onset times). In other words, changes in phase locking not only reflect changes in phase-concentrations with regard to the onsets of the auditory beats, but also changes in phase-concentrations with regard to the onsets of the stimuli relevant for memory encoding and retrieval.

### Statistical Analyses

Auditory beat stimulation conditions were compared to the control condition based on non-parametric label-permutation cluster statistics (Maris and Oostenveld, 2007), that correct for multiple comparisons across frequencies and time points. First, paired *t*-tests were conducted across all patients for each time point (-200 to 2000 ms). In a second step, neighboring time points with significant *t*-tests (*p* < 0.05) were clustered and the cluster-value was calculated as the sum of *t*-values within the cluster. For each cluster, cluster-values were compared to maximal cluster-values of label-permutated data. For this purpose, condition labels (beat vs. control) were permuted (31/63/127 possible permutations, corresponding to 5/6/7 contacts), and cluster-values were again calculated on the basis of paired *t*-tests for each permutation. Then, each cluster-value for the original data was ranked among the maximum cluster-values resulting from random label permutation to obtain the final *p*-value.

For significant intervals in the rhinal phase locking data, further analysis of iEEG phases was conducted to compare phase distributions for monaural and binaural beat conditions. For this purpose, trials were merged across all patients. To ensure equal weights the same number of trials was randomly chosen for each patient (23 per condition). The following analyses were performed 10 times each with a new subset of randomly chosen trials and results were averaged across all 10 calculations. First Rayleigh tests (function circ_rtest) were performed for phase values across all patients for each time point of the selected intervals. A significant Rayleigh test indicates that phases are not uniformly distributed but exhibit significant phase accumulations. To test for significant differences in phase distributions between binaural and monaural beat stimulation, non-parametric multi-sample tests for equal circular medians (function circ_cmtest), similar to Kruskal-Wallis tests for linear data, were conducted for each time point, for which the Rayleigh tests had indicated significant phase accumulations. All circular statistics were calculated using the free CircStat toolbox for MATLAB (Version 8.2, MathWorks Inc.; Berens, 2009).

For the time points with significant Rayleigh and Kruskal-Wallis tests, additional analyses were performed. Phase values were averaged across color trials (encoding) for each patient and each selected time point. Differences between mean phase values of all binaural vs. monaural beat trials were calculated. Rayleigh tests were performed with phase differences combined across all patients (6) and time points (58) to test for significant phase directions (i.e., non-uniform distributions of phase differences). Circular one-sample tests (function circ_mtest) similar to one-sample *t*-tests were conducted to test whether the mean directions of phase differences were different from zero, which would indicate significant differences between mean phases of binaural vs. monaural beat trials. The same analysis was performed to test for differences in mean phase values between binaural vs. control and monaural vs. control condition.

## Results

### Behavioral Responses

Recognition memory was significantly above chance as revealed by the probability of hits minus false alarms (correct minus incorrect old decisions; color: 57 ± 25%, *t*_12_ = 8.23, *p* < 0.001, scene: 51 ± 24%, *t*_12_ = 7.78, *p* < 0.001). Reaction times during recognition were significantly faster for remembered vs. forgotten words (remembered: 1.85 ± 0.42 s; forgotten: 1.97 ± 0.53 s; paired *t*-test *t*_12_ = -3.03, *p* = 0.0105). Probability for correct minus incorrect source recognition was also significantly above chance (color: 39 ± 22%, *t*_12_ = 6.24, *p* < 0.001, scene: 32 ± 25%, *t*_12_ = 4.61, *p* < 0.001). Reaction times were different for the three types of source responses (one-way repeated measures ANOVA, *F*_2,24_ = 14.82, *p* < 0.001; correct: 1.76 ± 0.42 s; incorrect: 1.90 ± 0.42 s, unsure: 2.18 ± 0.57 s) and exhibited a significant linear effect (correct < incorrect < unsure *F*_1,12_ = 17.18, *p* = 0.001). Pairwise comparisons of reaction times between conditions (paired two-tailed *t*-tests) yielded: correct vs. incorrect: *p* = 0.043; correct vs. unsure: *p* < 0.001; incorrect vs. unsure: *p* = 0.022.

**FIGURE 2:**
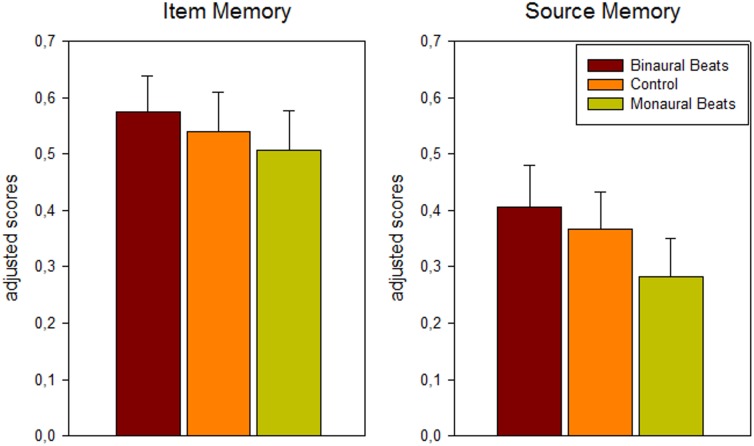
Behavioral results: dependence of memory scores on beat stimulation conditions. Bars show mean ± S.E.M. of adjusted scores for item (hits minus false alarms) and source (correct minus incorrect source association) memory across color and scene runs and across patients (*n* = 13).

A 3-way repeated-measures ANOVA (memory: item/source; association: color/scene; stimulation: binaural beat/monaural beat/control) revealed significant main effects for stimulation (*F*_2,24_ = 4.45; *p* = 0.03; Huynh-Feldt corrected) and association (*F*_1,12_ = 6.82, *p* = 0.023, color > scene), as well as the expected main effect for memory (*F*_1,12_ = 16.17, *p* = 0.002, item > source). There were no significant interactions between any of the three factors. For the factor stimulation a significant linear effect in the hypothesized direction was observed, indicating more adjusted hits and higher adjusted source memory under binaural beat vs. control vs. monaural beat stimulation (binaural > control > monaural; *F*_1,12_ = 5.59, *p* = 0.036; see Figure 2; adjusted hits (mean ± S.E.M.): binaural: 0.57 ± 0.07; control: 0.54 ± 0.07; monaural: 0.51 ± 0.07; adjusted source memory: binaural: 0.41 ± 0.07; control: 0.37 ± 0.07; monaural: 0.28 ± 0.07).

Pairwise comparisons between the stimulation conditions [paired one-tailed *t*-tests, effect size Hedges’ g corrected for sample size (Hedges and Olkin, 1985)] yielded for adjusted hits: binaural > monaural: *p* = 0.056, *g* = 0.27; binaural > control: *p* = 0.085, *g* = 0.13; control > monaural: *p* = 0.161, *g* = 0.13; for adjusted source memory: binaural > monaural: *p* = 0.024, *g* = 0.47; binaural > control: *p* = 0.162, *g* = 0.15; control > monaural: *p* = 0.015, *g* = 0.34.

### Phase Synchronization, Phase Locking and Power Values

**FIGURE 3:**
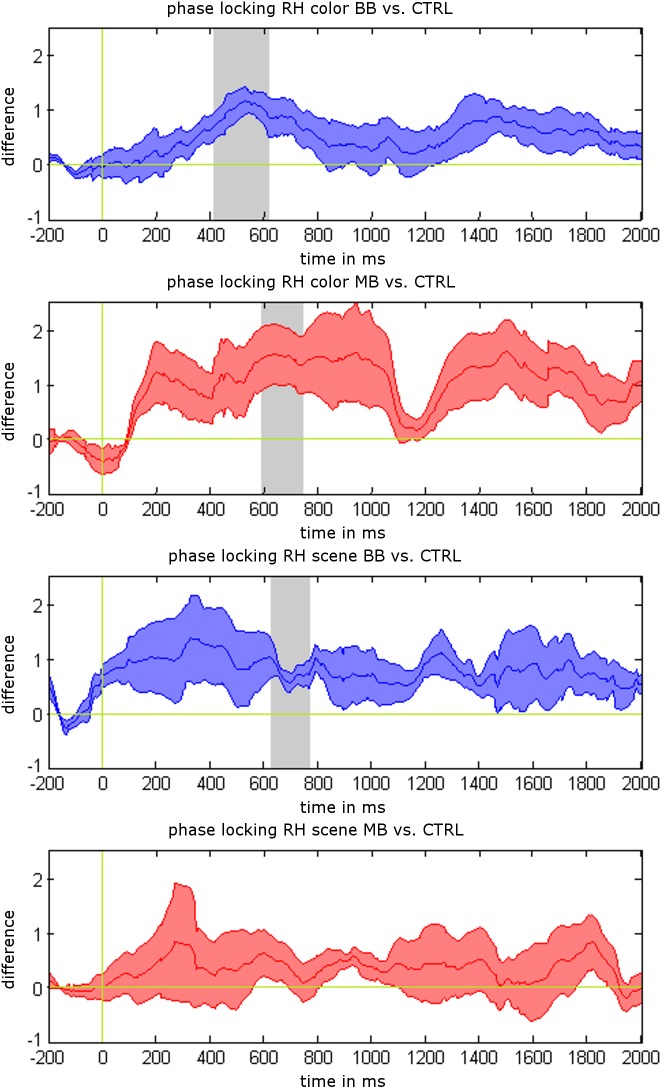
Phase locking results for 5 Hz beat stimulation vs. control condition. Mean phase locking differences in the rhinal cortex are shown for binaural beats (BB) vs. control (CTRL) and monaural beats (MB) vs. control condition. All responses are averaged across patients. Shaded areas indicate standard error of the mean (S.E.M.). Gray areas show significant time intervals in a label-shuffled cluster statistic for stimulation vs. control in each condition (*p* < 0.05).

We analyzed differences between beat stimulation and control conditions for iEEG power values, phase locking, and synchronization based on non-parametric label-permutation cluster statistics. All time points within stimulation trials were evaluated at the beat stimulation frequency of 5 Hz. This analysis revealed significant clusters indicating differences in phase locking between beat and control conditions within rhinal cortex. Phase locking values were higher for binaural beats vs. control during scene trials between 622 and 762 ms (*p* = 0.031), for binaural beats vs. control during color trials between 409 and 611 ms (*p* = 0.016), and for monaural beats vs. control during color trials between 595 and 738 ms (*p* = 0.047; see Figure 3). There were no significant clusters for power values or phase synchronization.

Additionally, we tested for differences of mean phase locking values averaged across the complete stimulation interval (0–2 s) between stimulation and control conditions (paired *t*-tests). Mean phase locking values in rhinal cortex were higher for binaural beats vs. control condition during color trials (*p* = 0.025) and scene trials (*p* = 0.032), and for monaural beats vs. control condition during color trials (*p* = 0.043). There were no significant differences for monaural beats vs. control during scene trials (*p* = 0.45), or for any of these contrasts in the hippocampus (each *p* > 0.10).

### Rhinal Phase Values

**FIGURE 4:**
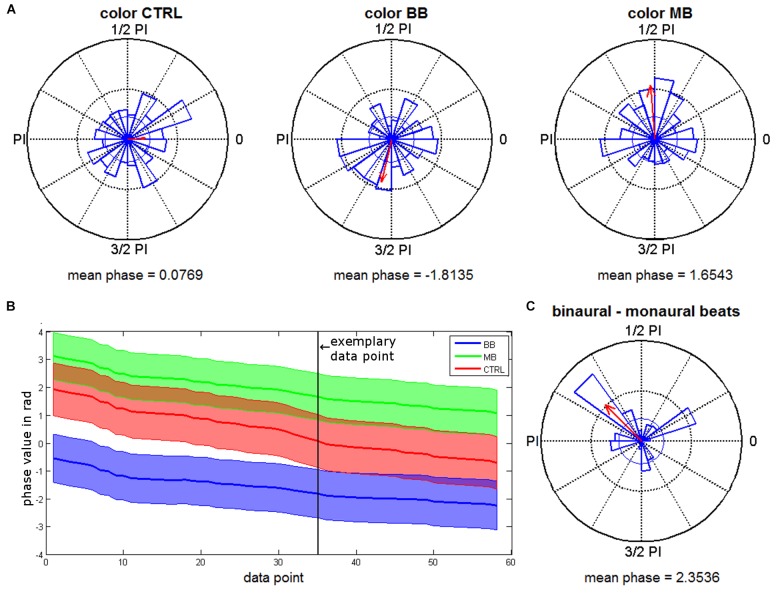
Phase distributions for the different beat stimulation conditions. **(A)** Phase distributions from a time point with significant Rayleigh and Kruskal-Wallis tests (663 ms). Red arrows give the mean resultant vector with a scaled length (the inner circle indicates the vector length corresponding to a *p*-value of 0.05). **(B)** Mean phase values of all time points with significant Rayleigh and Kruskal-Wallis tests for binaural (BB) and monaural (MB) beat conditions and control (CTRL) condition (encoding) are shown. Shaded areas indicate circular variance. The exemplary data point marked shows the time point chosen for plot in **(A)**. **(C)** Phase differences between binaural and monaural beats during encoding (time points with significant Rayleigh and Kruskal-Wallis tests).

For the merged time interval with significant phase locking effects (409–762 ms), the distributions of rhinal phase values for the different stimulation conditions were further analyzed. Rayleigh tests were conducted for each time point to test for significant phase accumulations. As expected based on the phase locking results, we found significant phase accumulations for time points within this interval (color binaural: 37.62%, color monaural: 49.41%, color control: 2.46%, scene binaural: 15.21%, scene monaural: 0.03%, scene control: 2.78%). Time points with significant phase accumulations for both beat stimulation conditions overlapped in 14.66% of the interval during color trials. There were no overlapping time points for scene trials. Next, based on the significant Rayleigh tests in both, monaural and binaural beat conditions, Kruskal-Wallis tests were performed for color trials to identify differences in average phase values between both conditions. 60.72% of the time points with significant Rayleigh tests showed significant differences between phase values for monaural vs. binaural stimulation (see Figure 4A for an exemplary time point and Figure 4B for all time points with significant Rayleigh and Kruskal-Wallis tests).

As suggested by the phase data that can be seen in Figures 4A,B, we finally examined whether rhinal phase values for binaural/monaural beats vs. control condition are significantly shifted toward opposite directions, which may represent phase values being optimal vs. detrimental for memory encoding. For all time points of color trials with significant Rayleigh and Kruskal-Wallis tests, differences of mean phase values were calculated between all binaural vs. monaural, binaural vs. control and monaural vs. control trials. Rayleigh tests revealed that all differences showed significant phase directions (each *p*-value < 0.02) and circular one-sample tests indicated significant deviations from zero for the phase differences between binaural vs. monaural (*p* < 0.001) and binaural vs. control condition (*p* < 0.001), as well as a trend for monaural vs. control condition (*p* = 0.085). The mean phase difference was 2.35 for binaural vs. monaural trials (see Figure 4C), 1.84 for binaural vs. control trials, and -0.67 for monaural vs. control trials. These findings indicate that rhinal phases are indeed shifted toward opposite directions by binaural, compared to monaural beat stimulation.

## Discussion

In this pilot study we investigated whether long-term memory can be modulated by monaural and binaural beat stimulation. In accordance with our hypothesis we observed that 5 Hz binaural stimulation enhanced, while 5 Hz monaural stimulation decreased memory performance in an associative learning task. Auditory beat stimulation had a similar impact on memory encoding and retrieval, as well as on item and source memory. However, the small sample size is a limitation of our study and non-significant interactions may be due to an insufficient statistical power to detect them. Compared to the control condition, both binaural and monaural stimulation were associated with phase locking increases of stimulus-related theta oscillations within rhinal cortex. These phase locking enhancements are likely due to entrainment of rhinal EEG oscillations with the beat stimuli. However, the phase locking increases for binaural and monaural stimulation corresponded to reverse phase shifts resulting in almost opposite phase values. Possibly, the different phase values are due to differences in sensory processing. For instance, steady-state responses to monaural beats may be triggered by signal peaks, whereas responses to binaural beats may be triggered when the intracranial sound image jumps from one ear to the other. Similar to the reported influence of phase values on neural communication (e.g., Womelsdorf et al., 2007) our data suggest that in our experiment binaural stimulation shifted rhinal phases toward values being optimal for memory processing, whereas monaural stimulation shifted phases toward detrimental values. Furthermore, our results are in line with previous findings indicating that rhinal engagement predicts both item and associative memory during encoding (Staresina and Davachi, 2008) and retrieval (Staresina et al., 2012).

Through which mechanism may rhinal phases affect memory processing? Rhinal phases most likely influence neural membrane potentials and thereby control firing thresholds via a mechanism of spike-field coupling (e.g., Elbert and Rockstroh, 1987). Such modulations of neural activity by field potential oscillations comparable to those measured *in vivo*, have been demonstrated *in vitro* and in simulations (e.g., Anastassiou et al., 2010; Fröhlich and McCormick, 2010). Thus, an optimal vs. detrimental rhinal phase may reflect whether neural activity occurs within the required time window or not. In this sense, rhinal memory operations such as semantic preprocessing and novelty detection may be facilitated or hampered. In line with these results, it has been shown that phases of human local field potentials in medial temporal regions code correct vs. incorrect matches in a card-matching task (Lopour et al., 2013). Furthermore, it has been demonstrated that successful verbal memory encoding can be predicted based on rhinal and hippocampal phase values (Höhne et al., 2016; Derner et al., 2018). With regard to scalp EEG similar findings have, for instance, been reported for auditory and visual perception of stimuli close to the detection threshold (e.g., Busch et al., 2009; Mathewson et al., 2009; Neuling et al., 2012).

The present finding indicating that stimulus-related phase locking of EEG activity within the rhinal cortex has an effect on long-term memory is in line with previously reported data. A study based on intracranial EEG data from 31 epilepsy patients addressed the capability of power, phase-synchronization and phase locking based mediotemporal EEG measures, in predicting long-term memory (Fell et al., 2008). One major outcome of this study was that stimulus-related rhinal phase locking measures were the best predictors of long-term memory (see Table 3 in Fell et al., 2008). However, in that study, we did not investigate the mean values around which phases were accumulated. In subsequent studies, we have demonstrated, based on machine-learning techniques, that memory formation can be successfully predicted based on rhinal and hippocampal single-trial phase values (Höhne et al., 2016; Derner et al., 2018). In particular, we have shown that memory performance can be predicted based on single-channel phase values from rhinal cortex (Höhne et al., 2016; Derner et al., 2018). We would like to clarify that this does not mean that only the presence of a certain optimal phase value at a certain time point is predictive for remembering, but that depending on the chosen time point within a larger range different phase values are predictive for remembering (for instance, a value of½ ^∗^ PI at 400 ms and a value of 3/2 ^∗^ PI at 500 ms, as well as other values at time points in between). This is reflected by the temporal extension of memory-related phase locking effects across several hundreds of milliseconds (Fell et al., 2008). Similar results have been reported by Lopour et al. (2013), with regard to a card-matching task. Taken together, these studies suggest that there are rhinal phase values which are optimal vs. detrimental for memory formation, and that an increased accumulation of rhinal phase values (as quantified by the phase locking measure) around certain mean values, can affect memory performance.

With regard to theta oscillations the notion of optimal phases does not necessarily refer to short sections of the theta cycle. In the present and in a previous study (Fell et al., 2008) we found that memory-related phase locking effects in the theta range were extended across periods on the temporal order of a theta cycle and above. That is, the term “optimal phase” refers to a wider range of phase values across the theta cycle. Indeed, there is evidence that the full theta cycle is utilized for phase coding of memories and that information is “chunked” across the theta cycle. For instance, it has been shown in rodents that location-selective neurons fire at specific theta phases, which can be distributed across the entire theta cycle (e.g., Colgin, 2013). It has been suggested that this so-called theta-phase precession is being generated in the entorhinal cortex and that it may represent a general coding mechanism underlying the formation of memories, in particular associative memories (Yamaguchi et al., 2007). Thus, binaural vs. monaural beat stimulation may modulate memory performance by shifting the phase of the rhinal theta cycle, such that different theta phases prevail when visual information arrives at the entorhinal-hippocampal system. Based on this interpretation, an interesting question for a future study would be whether the detrimental effect of monaural beat stimulation can be reversed by shifting the stimulation signal by radian PI (i.e., a half-cycle/180 degrees).

It has been demonstrated that monaural beats cause stronger percepts than binaural beats (e.g., Grose et al., 2012). However, we would like to add that this does not necessarily translate into stronger, but otherwise similar electrophysiological effects for monaural vs. binaural beats. Recent research indicates that EEG effects of binaural beats are to some extent qualitatively different from the effects of monaural beats, i.e., EEG effects differ depending on the brain region in which they were found (see Becher et al., 2015: for instance the following EEG effects were detected: a highly significant mediotemporal power decrease for 5 Hz monaural, but no decrease for 5 Hz binaural beats; a highly significant temporo-lateral phase synchronization increase for 5 Hz binaural, but no increase for 5 Hz monaural beats). It may be suggested that the observed behavioral effects are due to the capturing of attention by the beat stimulation (compared to stimulation using a pure sine wave), which may divert attention from the processing of memory-relevant stimuli. We would like to note that in this case it would be expected that both binaural and monaural beats would yield a decrease in memory performance, with a stronger decrease for monaural beats (due to the stronger percept). Hence, the linear effect for memory performance observed in our study (binaural > control > monaural) cannot be explained by such a mechanism.

It is indeed very difficult to prove the generalizability of iEEG results obtained from epilepsy patients to healthy subjects, since a direct comparison between iEEG recorded from epilepsy patients and healthy controls is not feasible. However, it has been shown that iEEG data obtained from the non-pathological MTL in patients with unilateral seizure origins during auditory and visual oddball experiments are qualitatively similar to those iEEGs recorded in healthy monkeys (Paller et al., 1992). These findings go some way to support the validity of our approach. Still, we cannot completely exclude an influence of epilepsy pathophysiology on our findings. It has, for instance, been reported that the performance of patients with temporal-lobe epilepsy in a sound-lateralization task is impaired when compared to healthy controls (Tezer et al., 2012). This finding implies that we cannot be absolutely sure that binaural beats are processed identically in patients with temporal lobe epilepsy as in healthy controls.

In an earlier study Ortiz et al. (2008) investigated the effect of binaural 5 Hz stimulation on verbal memory and reported enhanced task performance, which is in agreement with our findings. In their study, binaural stimulation was applied during the encoding phase of the memory paradigm. In contrast to these and our findings, a recent study has reported a detrimental effect of 5 Hz binaural beat stimulation on verbal memory (Garcia-Argibay et al., 2017). Furthermore, a study applying 7 Hz binaural beats has detected a beat stimulation-related decrease in immediate verbal memory recall, as measured by the Rey Auditory Verbal Learning Test (Wahbeh et al., 2007). As a major difference to the study design of Ortiz et al. (2008) and to our design, in these studies beat stimulation was applied before and not during the memory task. Hence, these findings tentatively suggest that the timing of beat stimulation is crucial for the direction of memory effects, an idea which is in line with the findings from a recent meta-analysis (Garcia-Argibay et al., 2018). The rhinal phase adjustments observed in our study, however, probably depend on instantaneous beat stimulation, which may account for the different behavioral outcome. In conclusion, our results suggest opposite effects of binaural vs. monaural beat stimulation on long-term memory, which are related to reverse phase adjustments.

## Data Availability

Data supporting the results can be made available upon reasonable request.

## Author Contributions

BS and JF designed the study. RS supervised the intracranial recordings. LC and MD acquired the data and analyzed the behavioral data. MD and JF analyzed the iEEG data. MD, LC, RS, BS and JF wrote the manuscript.

## Conflict of Interest Statement

The authors declare that the research was conducted in the absence of any commercial or financial relationships that could be construed as a potential conflict of interest.

## Abbreviations

EEG: electroencephalography
HI: hippocampal
iEEG: intracranial EEG
RH: rhinal
